# Process evaluation within pragmatic randomised controlled trials: what is it, why is it done, and can we find it?—a systematic review

**DOI:** 10.1186/s13063-020-04762-9

**Published:** 2020-11-09

**Authors:** Caroline French, Hilary Pinnock, Gordon Forbes, Imogen Skene, Stephanie J. C. Taylor

**Affiliations:** 1 Institute of Population Health Sciences, Barts and the London School of Medicine and Dentistry, Queen Mary University of London, 58 Turner Street, London, E1 2AB UK; 2 Usher Institute, The University of Edinburgh, Doorway 3, Medical School, Teviot Place, Edinburgh, EH8 9AG UK; 3 Institute of Psychiatry, Psychology and Neuroscience (IoPPN), Kings College London, 16 De Crespigny Park, London, SE5 8AF UK; 4 Emergency Department, Royal London Hospital, Barts Health NHS Trust, Whitechapel, London, E1 1FR UK

**Keywords:** Process evaluation, Pragmatic randomised controlled trials, Health services research

## Abstract

**Background:**

Process evaluations are increasingly conducted within pragmatic randomised controlled trials (RCTs) of health services interventions and provide vital information to enhance understanding of RCT findings. However, issues pertaining to process evaluation in this specific context have been little discussed. We aimed to describe the frequency, characteristics, labelling, value, practical conduct issues, and accessibility of published process evaluations within pragmatic RCTs in health services research.

**Methods:**

We used a 2-phase systematic search process to (1) identify an index sample of journal articles reporting primary outcome results of pragmatic RCTs published in 2015 and then (2) identify all associated publications. We used an operational definition of process evaluation based on the Medical Research Council’s process evaluation framework to identify both process evaluations reported separately and process data reported in the trial results papers. We extracted and analysed quantitative and qualitative data to answer review objectives.

**Results:**

From an index sample of 31 pragmatic RCTs, we identified 17 separate process evaluation studies. These had varied characteristics and only three were labelled ‘process evaluation’. Each of the 31 trial results papers also reported process data, with a median of five different process evaluation components per trial. Reported barriers and facilitators related to real-world collection of process data, recruitment of participants to process evaluations, and health services research regulations. We synthesised a wide range of reported benefits of process evaluations to interventions, trials, and wider knowledge. Visibility was often poor, with 13/17 process evaluations not mentioned in the trial results paper and 12/16 process evaluation journal articles not appearing in the trial registry.

**Conclusions:**

In our sample of reviewed pragmatic RCTs, the meaning of the label ‘process evaluation’ appears uncertain, and the scope and significance of the term warrant further research and clarification. Although there were many ways in which the process evaluations added value, they often had poor visibility. Our findings suggest approaches that could enhance the planning and utility of process evaluations in the context of pragmatic RCTs.

**Trial registration:**

Not applicable for PROSPERO registration

## Background

There are increasing calls for process evaluations alongside outcome evaluations of complex healthcare interventions [1–3]. Defining features of ‘complex interventions’ include having multiple interacting components, addressing multiple outcomes, and targeting different levels of change within complex systems [4]. Process evaluations increase understanding of complex healthcare interventions by studying aspects of implementation, mechanisms of impact, and context [4]. They may thus shed light on the ‘black box’ of complex interventions and provide information to interpret outcome results and aid implementation into practice [4, 5]. There has been similar increasing interest in the use of pragmatic randomised controlled trials (RCTs) to evaluate the outcomes of complex healthcare interventions [1, 6]. Pragmatic RCTs, in contrast to explanatory RCTs, aim to conduct ‘real-world’ evaluation of interventions, with findings that have enhanced generalisability to real-world clinical practice [6].

Masterson-Algar et al. [7] highlight the importance of tailoring process evaluation guidance to the context in which it will be used, and accordingly, this review aims to address gaps in knowledge about process evaluation conduct in the context of pragmatic RCTs of health services interventions. The UK Medical Research Council (MRC) published comprehensive guidance for designing and conducting process evaluations of complex interventions in 2014 [4], following earlier process evaluation frameworks by other authors [5, 8, 9]. However, apart from Grant et al.’s framework [5], these were developed primarily for public health research. Although being described as applicable to health services research, many of the examples in the MRC’s guidance [4] are from a public health perspective. It is therefore useful to review process evaluation conduct in health services settings as these are likely to present some unique challenges. The few published systematic reviews of process evaluation methodology focus on specific fields of clinical practice [10–15] rather than outcome evaluation methods. The pragmatic RCT method is not explicitly addressed in existing process evaluation guidance, although some pertinent methodological issues are discussed, for example avoiding Hawthorne effects from patients participating in process evaluation interviews [4]. Nonetheless, concerns have been raised relating to pragmatic RCTs, such as the potential variability of usual care within control groups, and the potential impact of interventions beyond intervention recipients, such as to carers and family members [16]. Process evaluations present opportunities to examine and address such issues.

This review aims to provide insight into the state of process evaluation in the context of pragmatic RCTs in health services research, along with the reported value, barriers, and facilitators to conducting them. We also examine two issues identified as problematic, both from our own experience and within the process evaluation literature. Firstly, we investigate labelling, as the label ‘process evaluation’ has been applied to many types of study [4], and previous reviews noted inconsistent use of the term [5, 10]. We have also anecdotally encountered confusion and multiple interpretations of the meaning of the label. Secondly, we examine accessibility as suboptimal reporting has been highlighted, such as time delay and poor linkages between trial and process evaluation results publications [4].

Our aims were, within a systematically identified sample of published pragmatic health services research RCTs, to:

1. Describe the process data reported in trial results papers
2. Describe the frequency of separate process evaluation publications
3. Describe the use of the label ‘process evaluation’
4. Describe the characteristics of process evaluations
5. Synthesise reported practical barriers and facilitators to process evaluation conduct
6. Synthesise the reported values of the process evaluations
7. Describe the accessibility of process evaluation results

## Methods

**Fig. 1:**
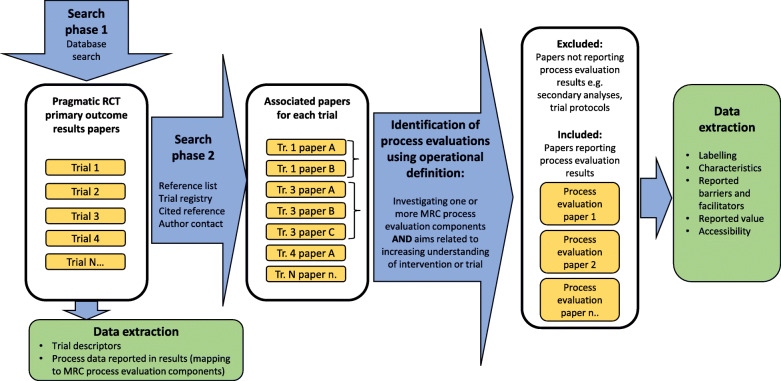
Method overview

Similar to previous systematic reviews of process evaluations [11, 12], we used a 2-phase search process. We firstly systematically identified an index sample of journal articles reporting the primary outcome results of pragmatic RCTs evaluating health services interventions (hereafter referred to as ‘trial results papers’) and then systematically searched for all associated publications. Using an operational definition of process evaluation based on the MRC’s framework [4], we then identified the process evaluations reported in associated publications, regardless of how they were labelled. We also identified any process data reported in index trial results papers which mapped to MRC process evaluation components. Figure 1 illustrates the methods, and Table 1 shows the MRC process evaluation components.

**Table 1:** MRC process evaluation components (adapted from [4], with definitions in italics where provided in original)

**Context**		
** Causal mechanisms present within the context** that act to maintain the status quo, or enhance effects	**Contextual factors** that shape theory of how the intervention works	**Contextual moderators** *Shape, and may be shaped by, implementation, intervention mechanisms, and outcomes*
**Implementation**		
** Dose** * How much intervention is delivered*	**Fidelity** *The consistency of what is implemented with the planned intervention*	**Adaptations** *Alterations made to an intervention in order to achieve better contextual fit*
** How delivery is achieved** * The structures, resources and mechanisms through which delivery is achieved*	**Reach** *Extent to which target audience comes into contact with intervention*	
**Mechanisms of impact**		
** Mediators** * Intermediate processes which explain subsequent changes in outcome*	**Participant responses** *How participants interact with a complex intervention*	**Unanticipated pathways and consequences**

### Search strategy and inclusion and exclusion criteria

In the first search phase, we systematically identified an index sample of pragmatic RCTs. We limited the search to a single year, 2015 (selected to allow time for related publications to appear), and to MEDLINE Core Clinical Journals to provide a feasible number of papers. We searched MEDLINE (Ovid), and the full search strategy is given in Additional file 1.

#### Phase 1 inclusion criteria (PICOS)

- Population: any
- Intervention: any delivered by a health service
- Comparator: any
- Outcome: any
- Study: pragmatic randomised controlled trial (defined as the use of the word ‘pragmatic’ to describe the RCT in the title or abstract)

#### Phase 1 exclusion criteria

1. Papers not reporting the primary trial outcome
2. RCTs labelled as pilot, feasibility, or implementation studies
3. Trials of health interventions not delivered within health services, for example by charities

In phase 1, two reviewers (CF and IS) independently screened titles and abstracts against the inclusion and exclusion criteria, obtaining full texts as necessary. Any disagreements were discussed with ST and HP to reach a final decision on inclusion.

In phase 2 (see Fig. 1), citation searches for each trial results paper were conducted using both Web of Science (Clarivate Analytics) and Google Scholar. Corresponding authors were sent one reminder if we received no reply following the first contact. The searches were originally conducted, and authors contacted, in March and April 2018. Search phase 2 was updated in December 2019 apart from author contact.

We used an operational definition of ‘process evaluation’ to identify papers for inclusion regardless of how they were labelled by the study authors. As shown in Fig. 1, included studies investigated one or more MRC process evaluation components and (to distinguish them from trial secondary analyses or sub-studies) were aimed at increasing understanding of the intervention or trial. One reviewer (CF) screened all publications and discussed all considered to possibly be process evaluations with HP and ST in a consensus meeting to agree the final sample of process evaluations.

Several index trials were funded by the UK National Institute for Health Research’s *Health Technology Assessment* (HTA) programme. This programme requires results to be published as a monograph in the *Health Technology Assessment* journal, additional to any other journal publications. We therefore reviewed the full texts of all HTA monographs to check for process evaluation results.

### Data extraction and analysis

As this was a review of methodology rather than findings, we did not conduct any appraisal of quality of the included process evaluation studies. We extracted quantitative data to an Excel database and conducted descriptive analysis using SPSS v25. We extracted qualitative data as sections of text from PDFs of publications and used NVivo v11 for data management and to aid thematic analysis.

Where the methods or results from a single trial or process evaluation were reported in more than one publication (e.g. HTA monograph and separate journal paper), we extracted all available data from all publications but treated the publications as a single case. CF extracted and analysed all data independently, apart from the MRC process evaluation components as detailed below.

### Data extracted from the trial results papers

We extracted descriptors of all trials, and the data fields and their operationalisation are shown in Additional file 2. We mapped data items reported in the results sections to the MRC process evaluation framework [4] (see Table 1) to identify process data within the trial results papers. For example, a trial flow diagram (process data item) mapped to the process evaluation component ‘reach’. For each trial, we recorded whether each process evaluation component was reported in the trial results paper at least once. We piloted this process, and as the MRC guidance does not provide clear definitions for some components, we made a list of the types of data which mapped to each component (for example subgroup analyses mapped to ‘contextual moderators’). Three reviewers (CF, GF, and IS) independently extracted data from the first three trials, compared results, and agreed initial mappings. We used these to extract data from four further trials and again compared and discuss findings. CF then extracted data for the remaining trials, discussing any new mappings or uncertainties with the other authors.

### Data extracted from process evaluation publications

Table 2 shows the outcomes extracted for each process evaluation publication. O’Cathain et al. [17] noted that the value of qualitative research within RCTs is often not clearly articulated in publications, and we noted the same during scoping this review. We therefore operationalised ‘reported value’ as any reported rationales for undertaking a process evaluation, or any reported implications of having undertaken it or of its findings. This allowed us to capture any anticipated or observed benefits of the process evaluation or use of the knowledge it produced.

**Table 2:** Data outcomes for process evaluation publications

Review objective	Type of data	Outcomes
**Labelling**	Quantitative	• Use of label ‘process evaluation’ anywhere in the set of papers for the trial • Use of keyword ‘process evaluation’ for indexing
**Characteristics**	Quantitative	• Process evaluation components (mapped from aims and qualitative findings) • Whether processes related to the intervention or trial • Methodology • Data collection method
**Reported barriers and facilitators**	Qualitative	• Practical issues relating to designing or operationalising the process evaluation
**Reported value**	Qualitative	• Reported rationales for undertaking, or implications of the process evaluation
**Accessibility**	Quantitative	• Publishing journal • Time to publication from trial results paper • Search method required to locate paper • Mention of the process evaluation in trial results paper • Where in paper the trial first named or referenced • Inclusion in trial registry

A completed PRISMA checklist is in Additional file 3.

## Results

**Fig. 2:**
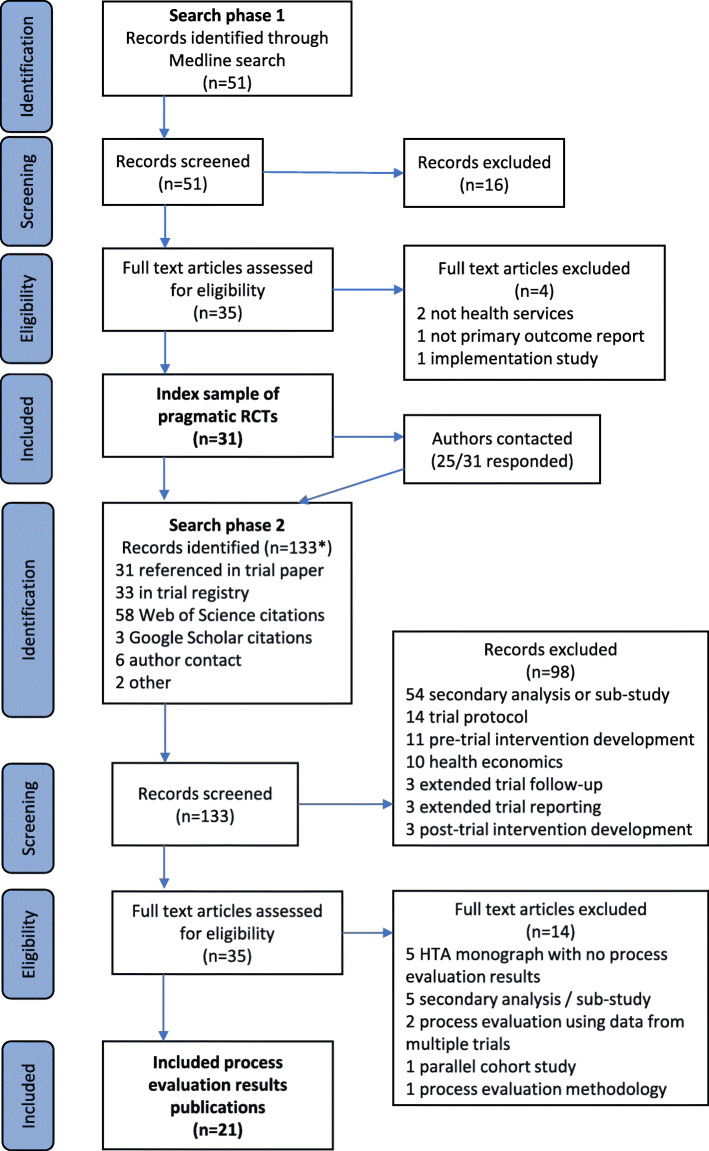
Adapted PRISMA flow diagram [18]. The asterisk indicates searches conducted in order stated and each record included only under search method first found

Figure 2 shows the results of search phases 1 and 2. The first search phase yielded 31 journal articles reporting primary outcome results from pragmatic RCTs, and the second phase located 133 associated publications. We categorised 21 of these 133 associated publications as process evaluation results. These covered 17 separate process evaluation studies, as some were published in more than one paper.

### Characteristics of the sample of pragmatic RCTs

The sample of pragmatic RCTs (*n* = 31) was highly variable in terms of intervention and trial characteristics (see Additional file 4 for details of the RCTs). They covered 20 different clinical specialties and 17 different combinations of professionals involved in intervention delivery. Most interventions (28/31) were received by patients only, with the remainder directed at staff or staff and patients. Table 3 summarises further characteristics of the included trials.

**Table 3:** Characteristics of the index sample of pragmatic RCTs

**Randomisation level**		**Comparator**	
Individual	25	Usual care	15
Cluster	6	Other intervention(s)	10
		Stepped-wedge control period	2
**Design**		Comparing two settings	1
2-arm	22	Comparing two deliverers	1
Non-inferiority (2-arm)	4	No intervention	1
3-arm	3	Sham clinical procedure	1
Crossover	1		
Stepped-wedge	1	**Publishing journal**	
		British Medical Journal	7
**Primary outcome result**		Lancet	7
No evidence of effect	15	JAMA	5
Evidence of effect	11	Canadian Medical Association Journal	2
Non-inferiority trial	4	JAMA Pediatrics	2
Unclear	1	Critical Care Medicine	1
		Gut	1
**Funder**		JAMA Internal Medicine	1
Public	25	JAMA Psychiatry	1
Multiple funders	3	Journal of Allergy and Clinical Immunology	1
Charity	1	New England Journal of Medicine	1
Independent Organisation	1	Nursing Research	1
Not reported	1	The American Journal of Psychiatry	1
**Type of intervention**		**Country**	
Pharmacological treatment strategy	9	UK	12
Clinical procedure	4	USA	8
Therapy intervention	4	Australia	3
Clinical treatment strategy	3	Netherlands	2
Model of care provision	3	Brazil	1
Reminder system	3	Canada	1
Health promotion	3	France	1
Medical device	2	France, Belgium and Switzerland	1
		Hong Kong	1
		North America^a^	1

^a^Countries not specified in original article

### Process evaluations

Twelve of the 31 pragmatic RCTs had at least one associated publication which we classified as reporting process evaluation results. We identified 17 distinct process evaluation studies, with two trials [19, 20] having three process evaluations and one trial [21] having two process evaluations. Although it is likely that these multiple process evaluation studies in the same trials formed part of one overall process evaluation, as each was presented as a distinct study, we extracted data from each individually. The 17 process evaluation studies were published across 21 publications, as some were published in both a journal article and HTA monograph.

The 17 process evaluation studies are listed in Table 4.

**Table 4:** Included process evaluation studies

Reference(s)	Description of process evaluation	Methodology and data collection methods	Intervention or trial processes	Process evaluation components	Labelled as process evaluation
Ball 2018 [22]	Investigated the effect of mild cognitive impairment in participants on intervention outcome	Quantitative Trial dataset	Intervention	Contextual moderators	No
Clark 2015 [23]	Explored patient perceptions of the acceptability of intervention in both groups, and motivations for agreeing or refusing to participate in the trial	Qualitative Interviews	Intervention and trial	Participant responses Reach Contextual moderators Unintended consequences Causal mechanisms in context	No
Grubbs 2015 [24]	Investigated which factors predicted patient uptake of an element of the intervention found to mediate the primary outcome	Quantitative Medical record review	Intervention	Contextual moderators	No
Handoll 2016 [25]	Described how the intended fracture population was practically achieved in pragmatic RCT, including results of formal independent assessment and classification of trial fractures	Quantitative Detailed author description Trial dataset	Intervention and trial	Reach	No
Handoll 2015 [26]					
Handoll 2014 [27]	Described processes undertaken to ensure usual care received by both groups in trial was good quality and comparable, including results of methods described	Quantitative Detailed author description Deliverer self-report	Intervention and trial	How delivery is achieved Fidelity	No
Handoll 2015 [26]					
Hall 2017 [28]	Investigated mediators of intervention outcome	Quantitative Trial dataset	Intervention	Mediators	No
Hill 2016 [29]	Explored perceptions of ward staff about how intervention contributed to outcome, and experience of intervention being delivered on their ward	Qualitative Focus groups	Intervention	How delivery is achieved Participant responses Contextual moderators Causal mechanisms in context Contextual factors shaping intervention theory	No
Hill 2016 [30]	Explored patient experiences of intervention and perceived barriers to engagement	Qualitative Semi-structured questionnaires	Intervention	Participant responses Causal mechanisms in context Contextual factors shaping intervention theory	Yes
Hill 2015 [31]	Explored perceptions of intervention deliverers of delivering intervention and how the intervention worked	Qualitative Focus groups, interview, field notes, intervention notes	Intervention	How delivery is achieved Contextual factors shaping intervention theory Participant responses Causal mechanisms in context	Yes
Keding 2019 [32]	Explored how patient and surgeon treatment preferences impacted recruitment, trial conduct, and patient outcomes	Quantitative Trial dataset	Intervention and trial	Reach Participant responses Contextual moderators	No
Handoll 2015 [26]					
Knowles 2015 [33]	Explored patient experiences of the intervention, including acceptability, ease of use, barriers to engagement, content, accessibility, and support. Also explored healthcare professional perceptions of feasibility and which patients’ intervention most suited to.	Qualitative Interviews	Intervention	Participant responses How delivery is achieved Reach Causal mechanisms in context Contextual moderators Unintended consequences Contextual factors shaping intervention theory	Yes
Littlewood 2015 [34]					
Nichols 2017 [35]	Explored experiences of patients about intervention, with focus on patient adherence, and how changed over time	Qualitative Interviews (longitudinal)	Intervention	Participant responses Causal mechanisms in context Contextual moderators How delivery is achieved	No
Williams 2015 [36]					
Novak 2015 [37]	Investigated whether and how trial sites supplied thawed plasma in a timely manner	Quantitative Detailed author description Observation, reports from sites	Intervention and trial	Fidelity How delivery is achieved	No
Sands 2016 [38]	Explored how the flexible complex intervention was delivered in real-world complex settings	Qualitative Trial dataset	Intervention	How delivery is achieved Adaptations Contextual moderators Participant responses Unintended consequences Contextual factors shaping intervention theory Fidelity	No
Saville 2016 [39]	Explored preferences and experiences of intervention deliverers about various aspects of intervention	Quantitative Questionnaire	Intervention	How delivery is achieved	No
Tjia 2017 [40]	Investigated patients’ perceptions of benefits and drawbacks of intervention	Quantitative Questionnaire	Intervention	Participant responses	No
Vennik 2019 [41]	Explored views and experiences of parents and practice nurses of intervention and usual care	Qualitative Interviews	Intervention	Participant responses How delivery is achieved Contextual factors shaping intervention theory Causal mechanisms in context Unintended consequences	No
Williamson 2016 [42]					

### Labelling

In the trial results papers, the label ‘process evaluation’ was never used to describe the process data. Five trials [19, 43–46] used variations of the labels ‘process outcome’ or ‘process measure’ for some data, although this use was infrequent and inconsistent.

Only three of the 17 studies we classified as process evaluations were labelled as process evaluations [30, 31, 33, 34]. One further study was not explicitly labelled as a process evaluation but this was implied as the MRC process evaluation guidance was cited as a rationale for undertaking it [28]. Only one of the three studies labelled as ‘process evaluation’ was clearly labelled as such in the article title [31]. One was described as ‘informing a process evaluation’ in the main article text [30]. The other was referred to as a process evaluation by the trial results paper [47], but not labelled as such in the journal article [33] or HTA monograph [34] reporting it.

Notably, one trial [19] had three qualitative studies published in the same journal: a qualitative interview study labelled as ‘a process evaluation’ [31], a qualitative questionnaire study reported as ‘informing the process evaluation’ [30], and a qualitative interview study labelled as a ‘qualitative evaluation’ [29]. However, the articles indicated that the studies were interlinked and formed a ‘sequential mixed-methods study’ [31].

None of the journal articles reporting process evaluation results (*n* = 16) used the keyword ‘process evaluation’.

### Characteristics of process evaluation studies

Of the 17 process evaluation studies identified, nine were quantitative [22, 24–28, 32, 37, 39, 40] and eight qualitative [23, 29–31, 34–36, 38, 41, 42]. The three labelled as process evaluations were all qualitative [30, 31, 33, 34]. There were a variety of data collection methods as can be seen in Table 4, with the use of trial data (*n* = 5), interviews (*n* = 4), and questionnaires (*n* = 3) being the most common. The reporting articles of three quantitative process evaluations [25, 27, 37] also presented detailed descriptions of trial or process evaluation methods.

Twelve process evaluations evaluated only intervention processes [22, 24, 28–31, 33–36, 38–42], and five evaluated both trial and intervention processes [23, 25–27, 32, 37]. Of the latter, one explored patients’ experiences of trial participation qualitatively [23] and two described in detail the trial processes undertaken to ensure fidelity [27, 37]. One investigated the trial processes for defining the pragmatic RCT trial population, by undertaking independent assessment of the radiographs used by recruiting surgeons to determine trial inclusion [25]. Another investigated the impact of surgeon and patient treatment preferences on trial recruitment and adherence to trial follow-up [32]. Further details of the processes evaluated by all 17 studies can be found in Table 4.

### Process evaluation components reported in the trial results papers and process evaluation papers

All 31 pragmatic RCTs reported process data in their trial results paper(s), with a median of five different MRC process evaluation components (IQR = 3; range 1–9) reported at least once per trial results paper. Further details can be found in Additional file 4.

**Fig. 3:**
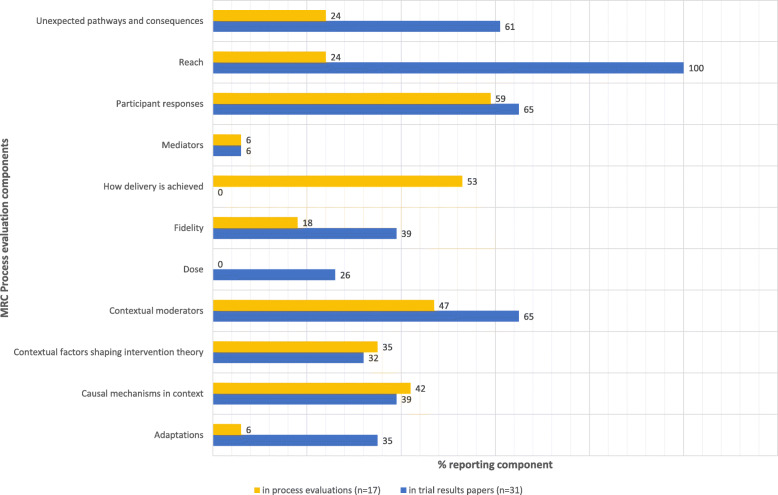
MRC process evaluation components reported in the trial results papers and process evaluations

Figure 3 shows the percentages of pragmatic RCTs (*n* = 31) reporting each MRC process evaluation component in their trial results paper(s) and the percentages of process evaluation studies (*n* = 17) reporting each component.

Although we found most of the identified process evaluation components to be reported in the main trial papers and/or in papers labelled process evaluations, the component ‘how delivery is achieved’ was only reported in process evaluation papers and ‘dose’ was only reported in trial results papers. The other ‘implementation’ components—‘fidelity’, ‘adaptations’, and ‘reach’ were more frequently reported in the trial results papers than the process evaluation papers.

Additional file 4 lists the included 31 pragmatic RCT results papers, and the process evaluation components reported in each. Additional file 5 shows the data items we mapped to each process evaluation component in the trial results papers and process evaluation papers.

### Barriers and facilitators to conducting process evaluations

**Fig. 4:**
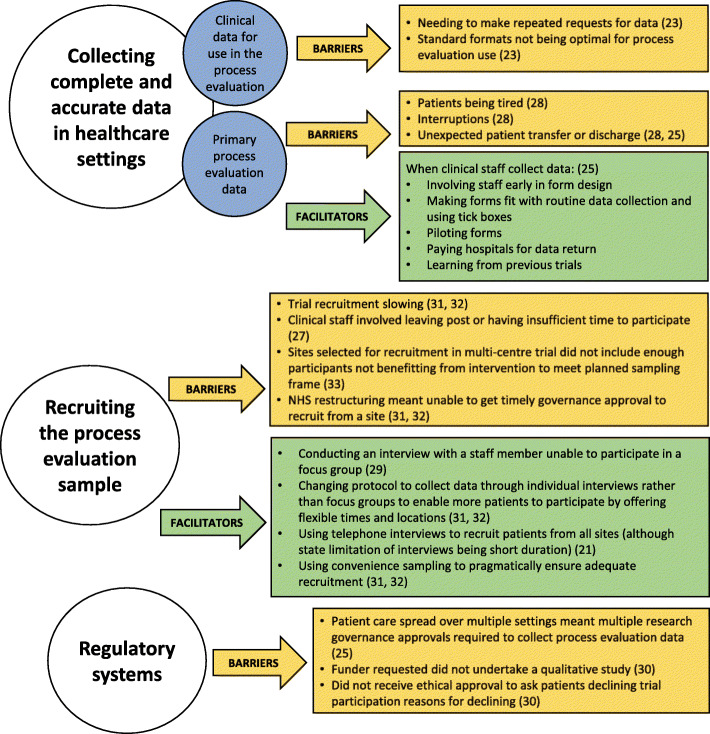
Reported barriers and facilitators

We identified three main themes of reported barriers and facilitators to conducting process evaluation within pragmatic RCTs, shown in Fig. 4. These themes were collecting complete and accurate data in health services settings, recruiting the process evaluation participants, and complex regulatory systems (only barriers identified within this theme).

### Reported value of the process evaluation studies

**Fig. 5:**
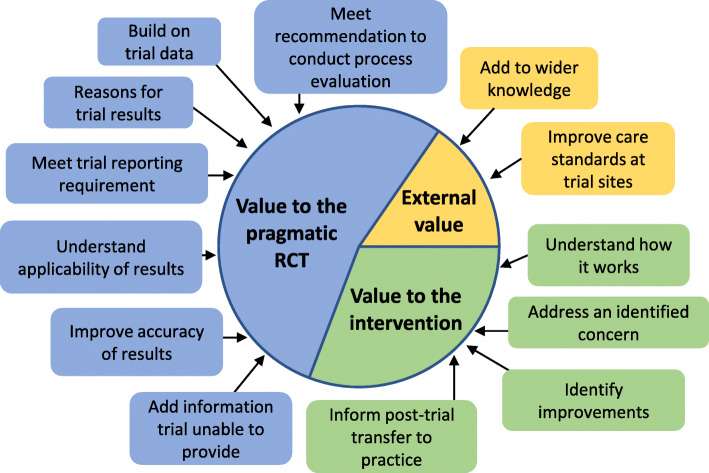
Synthesis of reported values of process evaluation studies

We identified three main themes relating to the reported value of the process evaluation: (1) whether the process evaluation added value to the intervention, (2) whether the process added value to the trial, or (3) whether the process evaluation’s value related to something external to the trial and intervention. Figure 5 shows the main themes and subthemes, and Table 5 shows the number of process evaluations mentioning each subtheme and examples of data relating to each subtheme. A full table of all data for each subtheme is in Additional file 6.

**Table 5:** Reported value subthemes

Subtheme	Number of process evaluations reporting this value (***n*** = 17)	Examples of reported values in subtheme
**Adding to wider knowledge**	16	• Informing future trial design [23, 25, 27, 28, 32, 33, 38] • Improving future design of similar interventions [22, 24, 33]
**Informing post-trial transfer of intervention to practice**	15	• Providing evidence of feasibility [28, 33] • Highlighting potential disadvantages of intervention to facilitate consent discussions with patients [23]
**Identifying intervention improvements**	10	• Adding stronger monitoring protocols to promote adherence [33] • Recommendation to research effectiveness over time [29]
**Providing reasons for trial results**	8	• Reasons for non-positive results [33, 38] • Reasons for positive results [28–31, 35]
**Addressing an identified concern about the intervention**	7	• Concern about the effect of cognitive impairment on effectiveness [22] • Concern about participant adherence [33, 35]
**Adding information not provided by the trial**	6	• Participant and deliverer experiences and perceptions [23, 35] • Nuance and context [23]
**Increasing accuracy of trial results**	6	• Investigating threats to internal validity [26] • Accurately defining trial population [25]
**Understanding how the intervention works**	4	• Understanding what was delivered in a flexible intervention [38] • Mechanisms of impact [28]
**Building on trial data**	2	• Exploring findings of subgroup analysis [29]
**Understanding applicability of trial results**	2	• Evaluating whether intended pragmatic trial population achieved [25]
**Improving usual care at trial sites**	1	• Highlighting gaps in current care provision [27]
**Meeting pragmatic RCT reporting requirements**	1	• Adhere to reporting standards for pragmatic and non-pharmacological trials [27]
**Meeting recommendation to conduct process evaluation**	1	• Following MRC recommendations [28]

### Reported value specifically relating to the pragmatic RCT

The reports of three process evaluations belonging to the same trial [25–27, 32] (not labelled as process evaluations) discussed the pragmatic nature of the trial and the process evaluations’ contributions in detail. All highlighted how they supported validity of trial results, by addressing potentially problematic areas of the pragmatic trial design. In one process evaluation [25, 26], the authors report it confirmed that the achieved trial sample was pragmatic as intended, and endorsed the pragmatic methods used to determine trial eligibility. In another [26, 27], the authors describe how it provided evidence of a good standard of, and therefore comparable, real-world clinical practice in the intervention and usual care delivered in the pragmatic trial across trial sites. In the final process evaluation [26, 32], the impact of patient and surgeon preference on internal and external validity is investigated, acknowledging that this is a threat to the validity of trial findings from the real-world setting.

No other reports explicitly discussed the pragmatic nature of the RCT. However, one process evaluation [38] used a qualitative content analysis to ‘describe the pragmatic reality’ of intervention delivery, and its authors emphasise that this was important to allow post-trial replication of a flexible intervention with a large potential variability of delivery in a complex setting. In the report of a qualitative interview study with intervention recipients and providers [42], the authors highlight that these process evaluation data provide real-life insights to aid post-trial implementation.

### Accessibility of process evaluation studies

Thirteen of the 17 process evaluation studies [22, 24, 28–32, 35–38, 40–42] had no mention in their corresponding index trial results papers.

Journal articles reporting process evaluation results (*n* = 16) were published a median of 15.5 months (range − 3–42; IQR 18.25) after the corresponding index trial results papers. None was published in the same journals as the trial results papers. Two trials had multiple process evaluation studies published in the same journals [25, 27, 29–31]. Twelve of the 16 process evaluation journal articles [22, 28–32, 35, 37–39, 41] were not included in the trial registry entries. A forward citation search of the index trial results paper was required to locate 9/16 of the process evaluation journal articles. Two process evaluation journal articles [37, 38] did not appear in the trial results paper, trial registry, or forwards citation searches. These were located by chance before contacting authors as they were mentioned in other papers associated with the trials. All process evaluation journal articles named or referenced the corresponding trial; however, 9/16 did not name or explicitly link it to the trial in the title or abstract [22, 24, 25, 29–31, 39–41].

Six of the 12 trials with process evaluation(s) were funded by the UK NIHR HTA programme and published an HTA monograph [23, 26, 34, 36, 42, 48]. One process evaluation was only reported in the HTA monograph [23], not a journal article. Six process evaluation studies were published at least in part in both a journal article [25, 27, 32, 33, 35, 41] and HTA monograph [26, 34, 36, 42]. Two process evaluations were part of HTA-funded trials; however, results were not reported in the HTA monographs, only in journal articles [28, 38].

The five HTA monographs reporting process evaluation findings [23, 26, 34, 36, 42] all appeared in the trial registry and were published a median of 1 month (IQR 3; range 0–4) after the trial results papers. Combining publication data for journal articles and HTA monographs therefore improved these aspects of accessibility for the whole sample of process evaluations (*n* = 17). If the earliest of the HTA monograph and journal article for each process evaluation is included, process evaluation studies (*n* = 17) were published a median of 5 months (range 0–36; IQR 15.5) after the trial results. Similarly, 9/17 process evaluations were published in a publication included in the trial registry entry.

## Discussion

### Summary of findings

We identified a range of reported benefits of process evaluations to the interventions they evaluated, to the associated pragmatic RCTs, and beyond to wider knowledge. Nonetheless, only approximately one third (12/31) of the pragmatic RCTs included in this review had published process evaluations. However, many data items were reported in trial results papers, which we mapped to MRC-defined process evaluation components. Very few (3/17) studies which we categorised as process evaluations were labelled as such, and the label was used inconsistently in those which did employ it. The 17 process evaluations utilised a variety of qualitative and quantitative methods and examined a wide range of process evaluation components, including trial processes. We identified several practical barriers and facilitators to their design and conduct and found visibility and accessibility of process evaluation results were often suboptimal. We now discuss these findings and draw recommendations, with a summary of recommendations presented in Table 6.

**Table 6:** Summary of recommendations

**Recommendations for process evaluation design**	• Consider the identified potential values of process evaluation within pragmatic RCTs and how these may be realised and articulated to stakeholders • We encourage debate about the meaning of the label ‘process evaluation’ and how it may be more consistently applied
**Recommendations for process evaluation conduct**	• Consider the identified barriers and facilitators and how to address these when conducting process evaluations in health services settings
**Recommendations for process evaluation dissemination**	• Ensure process evaluation publications are included in the trial registry entry • Ensure process evaluations are mentioned in journal articles reporting the parent trial, and consider adding this item to relevant CONSORT checklists • Ensure process evaluation publications name or refer to the parent trial in the title or abstract • Publish strategies for conducting successful process evaluations and addressing challenges in health services settings, such as to recruiting process evaluation participants and collecting data

### Value, inclusion, and definitions

In the design and evaluation of complex interventions, there is increasing recognition that process evaluations are necessary [2], and calls for their routine inclusion [1]. In support of this, we identified a wide range of ways in which process evaluations may add value to interventions and trials. Some of the values we identified resonate with previous reviews [10, 49], such as informing post-trial implementation of interventions into practice and contributing to wider knowledge. We also identified some less recognised, for example improving the standard of care at trial sites by exposing gaps in current care provision [27]. These findings are useful to researchers to aid reflection on the potential value of process evaluations, and articulation of this to stakeholders. We did not investigate whether the reported value of the process evaluations related to whether or not the associated trial showed evidence of effect; however, this would be useful to include in future reviews.

Our findings suggest that, at least in 2015, process evaluations were far from routine in the health services research context. Nonetheless, our mapping of process evaluation components to outcomes reported in the trial results papers suggests that process was considered, even if they did not publish a separate process evaluation paper. This leads us to question the definition of process evaluation. Our perception of a process evaluation is that it is more substantial than measuring a single process outcome; however, when extensive process data are reported within trial results, the distinction between ‘a process evaluation’ and this suite of process data is less clear.

Further need for definitional clarity is demonstrated by the paucity and inconsistency of use of the label ‘process evaluation’ in the 17 separate studies. This echoes a finding of a previous systematic review [10], which reported only 32 of 124 ‘process evaluations’ used the label—a similar proportion to the labelling in our studies.

The MRC guidance [4] states that there is no unified definition of process evaluation, and the theoretical scope laid out in process evaluation frameworks and guidance [4, 5, 8, 9] is very broad, encompassing many methods, areas of investigation, and scales of study. This wide variety of possible characteristics of process evaluation is likely to generate confusion and may explain the inconsistent use of the label. Furthermore, the MRC guidance [4] only discusses process evaluation of interventions; however, in common with other authors [5, 50–53], we identified the important role for process evaluation in evaluating trial processes, such as recruitment and patient experience of trial participation. We therefore believe simply repeating previous calls for clearer labelling [5] is insufficient and recommend further discussion about the meaning of the term ‘process evaluation’.

### Barriers and facilitators

We identified several barriers and facilitators to process evaluation researchers collecting optimal data, recruiting participants, and working within regulatory frameworks in the real-world health service contexts in which pragmatic RCTs operate. Several of these identified challenges and enablers are not addressed in the MRC guidance [4]; however, a previous systematic review [10] recommended monitoring and reporting process evaluation recruitment. We recommend researchers continue to share their experiences of challenges and successful strategies for conducting process evaluations in this context.

### Indexing and visibility

Process evaluations often had poor visibility through not being mentioned in trial results papers, and/or not included in trial registries. Furthermore, time delay to publication, not naming trials in titles or abstracts, and not labelling or indexing as process evaluations were significant barriers to locating articles in citation searches. Reporting guidance for process evaluations is available [4, 5], emphasising the importance of linking outcome and process evaluation papers. Our findings demonstrate the importance of following these recommendations, specifically that outcome results journal articles should mention that a process evaluation was undertaken, and process evaluation journal articles should name or explicitly link to the trial in their title or abstract. We additionally recommend process evaluation articles are included in trial registries and that mention of any process evaluation undertaken could usefully be added to relevant CONSORT trial reporting checklists [54, 55]. We also highlight that some HTA monographs reported process evaluations alongside trial outcomes and integrated discussion of findings [23, 26, 34, 36, 42], and therefore demonstrate a useful reporting format.

### Strengths and limitations

The key design strength of this review was using an index sample of pragmatic RCTs and then identifying any reported ‘process evaluation’ using an operational definition. This provided valuable information on process evaluation frequency and accessibility and highlighted the inconsistency of the use of the ‘process evaluation’ label. However, a limitation is that we could include only a sample of pragmatic RCTs. Limiting to trials published in MEDLINE *Core Clinical Journals* means findings are likely reflective of well-funded health services research trials but may not be representative of trials published elsewhere. We also only included RCTs described as ‘pragmatic’ in the title or abstract. As such labelling is not an essential reporting criterion for pragmatic RCTs [54], trials were not identified for inclusion if they only used the term ‘pragmatic’ elsewhere in the paper.

Limiting index trial inclusion to publication in 2015 ensured a reasonable length of time for publication of process evaluation papers, and indeed, two process evaluations were published in 2019. However, this also means findings may not be representative of process evaluations being designed and conducted now. Our findings can therefore only highlight potential areas of uncertainty, difficulty, or opportunity, with alternative research approaches such as surveys or interviews needed to examine current practice. We also acknowledge as a limitation that we used the MRC process evaluation framework to identify and describe process evaluations, when most process evaluations in our sample (associated with trials published in 2015) would very likely have been designed prior to publication of the MRC guidance [4].

The search methods for identifying associated publications were comprehensive, with a good response rate from authors. We used a robust process for deciding which publications to categorise as process evaluations, and the team included highly experienced health service researchers with experience of designing and conducting process evaluations. We acknowledge others may disagree with our operational definition and categorisations; however, we highlight this ambiguity is itself an important finding.

Double data extraction was carried out on fields we considered to be subjective, increasing the reliability of findings. There are currently no agreed quality assessment standards for process evaluations [4], and therefore, we did not appraise the quality of included studies; however, doing so would add to and strengthen the findings.

## Conclusion

This review provides valuable insight into the frequency and characteristics of process evaluations, within a sample of systematically identified index pragmatic RCTs published in a single year, and highlights challenges and enablers to their practical conduct in health services settings. Significantly, it suggests that the definition of process evaluation is inconsistent and that the meaning of the term requires clarification. Despite the wide range of identified values of process evaluations, this review highlights important problems with accessibility, which are likely barriers to fully realising this value. Often, process evaluations are invisible in pragmatic RCT reporting, and we therefore make several straightforward but significant reporting recommendations.

## Supplementary information

**Additional file 1.** MEDLINE (Ovid) search strategy.

**Additional file 2.** Trial descriptor data fields.

**Additional file 3.** PRISMA 2009 checklist. Completed PRISMA checklist.

**Additional file 4.** Included pragmatic RCTs. Details and references of the 31 index pragmatic RCTs.

**Additional file 5.** Items mapped to each process evaluation component.

**Additional file 6.** All extracted values of process evaluation.

## Data Availability

The datasets used and/or analysed during the current study are available from the corresponding author on reasonable request.

## Abbreviations

HTA: Health Technology Assessment (UK *National Institute of Health Research* funding programme)
MRC: Medical Research Council
RCT: Randomised controlled trial
