# Supplementary material for: Process evaluation within pragmatic randomised controlled trials: what is it, why is it done, and can we find it?—a systematic review
Source: Trials. 2020 Nov 9;21:916. doi: 10.1186/s13063-020-04762-9 (PMC7650157; doi:10.1186/s13063-020-04762-9)
Supplement: Supplementary file 1 — Additional file 1. MEDLINE (Ovid) search strategy. [file 13063_2020_4762_MOESM1_ESM.docx]

**Additional file 1**

**MEDLINE (Ovid) search strategy**

Search conducted via Ovid (MEDLINE)® and EPub Ahead of Print In-Process & Other Non-Indexed Citations, Daily and Versions®

| 1. (pragmatic and trial).mp. [mp=title, abstract, original title, name of substance word, subject heading word, keyword heading word, protocol supplementary concept word, rare disease supplementary concept word, unique identifier, synonyms] |
| --- |
| 2. limit 1 to (english language and humans and "core clinical journals (aim)" and yr="2015" and (clinical trial, all or clinical trial or controlled clinical trial or pragmatic clinical trial or randomized controlled trial)) |
