# Supplementary material for: Process evaluation within pragmatic randomised controlled trials: what is it, why is it done, and can we find it?—a systematic review
Source: Trials. 2020 Nov 9;21:916. doi: 10.1186/s13063-020-04762-9 (PMC7650157; doi:10.1186/s13063-020-04762-9)
Supplement: Supplementary file 2 — Additional file 2. Trial descriptor data fields. [file 13063_2020_4762_MOESM2_ESM.docx]

**Additional file 2 - trial descriptor data fields**

| **Data field** | **Operationalisation** | **Extract from** | **Extract as** |
| --- | --- | --- | --- |
| **Funder** | Who funded the trial | Trial results paper, or trial registry if not stated | Free text |
| **Publication month** | Month the trial results paper was published in print, or online for online only journals | Medline search result | Month |
| **Publication year** | Year the trial results paper was published in print, or online for online only journals | Medline search result | 2015 |
| **Country** | The country / countries the intervention was delivered in during the trial | Trial results paper | Country |
| **Journal** | The journal the trial results paper was published in | Trial results paper | Journal name |
| **Intervention** | Brief description of intervention(s) | Trial results paper | Free text |
| **Comparator** | What was received by the control / comparator group(s) | Trial results paper | Free text |
| **Intervention recipients** | Who received the intervention | Trial results paper | Free text |
| **Intervention deliverer** | Who administered / delivered the intervention(s) during the trial | Trial results paper | Free text |
| **Clinical specialty** | The clinical field the intervention was intended for | Trial results paper | Free text |
| **Setting** | The setting of intervention delivery. | Trial results paper | Free text |
| **Randomisation level** | Whether participants were individually or cluster randomised | Trial results paper | Individual  Cluster |
| **Primary outcome result** | Whether the primary outcome result was stated as being statistically significant in the abstract of the paper (p value or confidence interval) | Trial results paper – abstract  If not clear from abstract class as unclear  If classification does not fit e.g. non-inferiority, multiple outcomes, class as n/a | Positive  Not positive  n/a if does not fit |
| **Trial design** | Further details of the trial design | Trial results paper | 2-arm  3-arm  Non-inferiority  Stepped-wedge  Crossover |
