# Supplementary material for: Process evaluation within pragmatic randomised controlled trials: what is it, why is it done, and can we find it?—a systematic review
Source: Trials. 2020 Nov 9;21:916. doi: 10.1186/s13063-020-04762-9 (PMC7650157; doi:10.1186/s13063-020-04762-9)
Supplement: Supplementary file 4 — Additional file 4. Included pragmatic RCTs. Details and references of the 31 index pragmatic RCTs. [file 13063_2020_4762_MOESM4_ESM.docx]

**Additional file 4 - included pragmatic RCTs**

|  |  | **MRC process evaluation components reported in index trial results paper(s)** | | | | | | | | | | |  |
| --- | --- | --- | --- | --- | --- | --- | --- | --- | --- | --- | --- | --- | --- |
| **Reference of index trial results paper**  *Journal*  Country  Further references* | **Intervention** | **Reach** | **Fidelity** | **Dose** | **How delivery is achieved** | **Adaptations** | **Contextual moderators** | **Contextual factors that shape intervention theory** | **Causal mechanisms that maintain status quo or enhance effects** | **Mediators** | **Unanticipated pathways and consequences** | **Participant responses** | **Separate process evaluation paper(s)** |
| Bartels 2015 (1)  *American Journal of Psychiatry*  USA | Health promotion coaching for obesity in serious mental illness | Y |  |  |  |  | Y | Y | Y | Y |  | Y | No (included process evaluation but using data from multiple trials so excluded from review) |
| Bender 2015 (2)  *JAMA Pediatrics*  USA | Speech recognition telephone calls to improve adherence to child asthma treatment | Y |  |  |  |  | Y | Y |  |  |  | Y | No |
| Boulvain 2015 (3)  *Lancet*  France, Belgium, Switzerland | Induction of labour vs expectant management for large-for-date foetuses | Y |  |  |  |  |  |  |  |  |  |  | No |
| Cooper 2015 (4)  *BMJ*  UK  Clark 2015 (5) | Outpatient vs inpatient uterine polyp treatment | Y |  |  |  |  | Y |  | Y |  | Y | Y | Yes |
| Curtis 2015 (6)  *Canadian Medical Association Journal*  Canada | Ultrasound or near-infrared vascular imaging to guide peripheral intravenous catheterisation | Y |  |  |  |  | Y | Y |  |  |  |  | No |
| El-Khoury 2015 (7)  *BMJ*  France | Balance training to prevent fall-induced injuries | Y |  | Y |  |  |  |  |  |  | Y | Y | No |
| Fortney 2015 (8)  *JAMA Psychiatry*  USA | Telemedicine-based collaborative care for veterans with PTSD | Y | Y | Y |  | Y |  |  |  | Y |  | Y | Yes |
| Gilbody 2015 (9)  *BMJ*  UK  Littlewood 2015 (10) | Computerised cognitive behavioural therapy for depression | Y |  |  |  |  | Y |  | Y |  | Y | Y | Yes |
| Hill 2015 (11)  *Lancet*  Australia | Individualised falls-prevention education for hospital patients, with training and feedback for staﬀ | Y | Y | Y |  |  | Y | Y |  |  | Y | Y | Yes |
| Holcomb 2015 (12)  *JAMA*  North America  Baraniuk 2014 (13)  Zhu 2016 (14) | Comparison of 2 different ratios of blood products in patients with major trauma | Y | Y |  |  | Y |  |  |  |  |  |  | Yes |
| Honkoop 2015 (15)  *Journal of Allergy and Clinical Immunology*  Netherlands | Comparison of 3 treatment strategies targeting different levels of asthma control | Y | Y |  |  |  |  |  |  |  |  | Y | No |
| Hui 2015 (16)  *Gut*  Hong Kong | Comparison of medical and nurse endoscopists performing colonoscopy | Y |  |  |  | Y | Y |  |  |  |  | Y | No |
| Kempe 2015 (17)  *JAMA Pediatrics*  USA | Collaborative centralised reminder/recall system to increase immunisation rates in young children | Y |  |  |  | Y |  | Y |  |  | Y |  | Yes |
| Knowles 2015 (18)  *Lancet*  UK  Horrocks 2015 (19) | Percutaneous tibial nerve stimulation for treatment of faecal incontinence | Y | Y |  |  |  | Y |  |  |  | Y | Y | No |
| Kutner 2015 (20)  *JAMA Internal Medicine*  USA | Statin discontinuation in advanced life-limiting illness | Y |  |  |  |  |  |  |  |  | Y | Y | Yes |
| Lamb 2015 (21)  *Lancet*  UK  Williams 2015 (22) | Exercises to improve hand function in rheumatoid arthritis | Y | Y | Y |  | Y | Y | Y | Y |  | Y | Y | Yes |
| Moreira 2015 (23)  *Nursing Research*  Brazil | Nursing case management for patients with type 2 diabetes | Y |  |  |  |  | Y |  | Y |  |  |  | No |
| Moseley 2015 (24)  *JAMA*  Australia | Exercise programme for rehabilitation following ankle fracture | Y | Y |  |  |  | Y |  | Y |  | Y | Y | No |
| Mouncey 2015a (25)  *NEJM*  UK  Mouncey 2015b (26) | Early, Goal-Directed Resuscitation protocol for septic shock | Y | Y |  |  | Y | Y | Y | Y |  | Y |  | No |
| Noto 2015 (27)  *JAMA*  USA | Chlorhexadine bathing in intensive care units | Y | Y |  |  |  | Y |  |  |  |  |  | No |
| Perkins 2015 (28)  *Lancet*  UK  Gates 2017 (29) | Mechanical vs manual chest compression for out of hospital cardiac arrest | Y | Y |  |  |  | Y |  | Y |  | Y |  | No |
| Rangan 2015 (30)  *JAMA*  UK  Handoll 2015 (31) | Surgical vs non-surgical treatment for adults with displaced fracture of proximal humerus | Y | Y |  |  | Y | Y |  | Y |  | Y | Y | Yes |
| Sackley 2015 (32)  *BMJ*  UK  Sackley 2016 (33) | Occupational therapy for care home residents with stroke disability | Y |  | Y |  |  | Y | Y |  |  | Y |  | Yes |
| Scott 2015 (34)  *BMJ*  UK  Scott 2014 (35) | Tumour necrosis factor inhibitors versus combination intensive therapy with conventional disease modifying anti-rheumatic drugs | Y |  |  |  | Y |  | Y |  |  | Y | Y | No |
| Semler 2015 (36)  *Critical Care Medicine*  USA | Electronic sepsis evaluation and management tool in intensive care | Y |  | Y |  | Y | Y |  |  |  |  | Y | No |
| Smith 2015a (37)  *BMJ*  UK | Patient-controlled analgesia for patients in emergency department with pain from traumatic injuries | Y |  |  |  |  |  |  |  |  | Y | Y | No |
| Smith 2015b (38)  *BMJ*  UK | Patient-controlled analgesia for patients in emergency department with pain from non-traumatic abdominal injuries | Y |  |  |  |  |  |  |  |  | Y | Y | No |
| Stewart 2015 (39)  *Lancet*  Australia | Standard vs atrial-fibrillation specific management strategy | Y | Y | Y |  | Y |  |  | Y |  |  |  | Yes |
| Wechsler 2015 (40)  *JAMA*  USA | Anticholinergic vs long-acting β-agonist in combination with inhaled corticosteroids in black adults with asthma | Y |  |  |  | Y | Y |  |  |  | Y | Y | No |
| Westendorp 2015 (41)  *Lancet*  Netherlands | Preventive antibiotics in stroke | Y |  | Y |  |  | Y | Y | Y |  | Y |  | No |
| Williamson 2015 (42)  *Canadian Medical Association Journal*  UK  Williamson 2015 (43) | Nasal balloon autoinflation in children with otitis media with effusion in primary care | Y |  |  |  |  | Y |  | Y |  | Y | Y | Yes |

*If applicable - references of additional publications reporting trial results from which we extracted data on process evaluation components

**References**

1. Bartels SJ, Pratt SI, Aschbrenner KA, Barre LK, Naslund JA, Wolfe R, et al. Pragmatic replication trial of health promotion coaching for obesity in serious mental illness and maintenance of outcomes. American Journal of Psychiatry. 2015;172(4):344-52.

2. Bender BG, Cvietusa PJ, Goodrich GK, Lowe R, Nuanes HA, Rand C, et al. Pragmatic trial of health care technologies to improve adherence to pediatric asthma treatment: a randomized clinical trial. JAMA Pediatrics. 2015;169(4):317-23.

3. Boulvain M, Senat MV, Perrotin F, Winer N, Beucher G, Subtil D, et al. Induction of labour versus expectant management for large-for-date fetuses: a randomised controlled trial. Lancet. 2015;385(9987):2600-5.

4. Cooper NA, Clark TJ, Middleton L, Diwakar L, Smith P, Denny E, et al. Outpatient versus inpatient uterine polyp treatment for abnormal uterine bleeding: randomised controlled non-inferiority study. BMJ. 2015;350:h1398.

5. Clark TJ, Middleton LJ, Am Cooper N, Diwakar L, Denny E, Smith P, et al. A randomised controlled trial of Outpatient versus inpatient Polyp Treatment (OPT) for abnormal uterine bleeding. Health Technology Assessment 2015;19(61).

6. Curtis SJ, Craig WR, Logue E, Vandermeer B, Hanson A, Klassen T. Ultrasound or near-infrared vascular imaging to guide peripheral intravenous catheterization in children: a pragmatic randomized controlled trial. CMAJ Canadian Medical Association Journal. 2015;187(8):563-70.

7. El-Khoury F, Cassou B, Latouche A, Aegerter P, Charles MA, Dargent-Molina P. Effectiveness of two year balance training programme on prevention of fall induced injuries in at risk women aged 75-85 living in community: Ossebo randomised controlled trial. BMJ. 2015;351:h3830.

8. Fortney JC, Pyne JM, Kimbrell TA, Hudson TJ, Robinson DE, Schneider R, et al. Telemedicine-based collaborative care for posttraumatic stress disorder: a randomized clinical trial. JAMA Psychiatry. 2015;72(1):58-67.

9. Gilbody S, Littlewood E, Hewitt C, Brierley G, Tharmanathan P, Araya R, et al. Computerised cognitive behaviour therapy (cCBT) as treatment for depression in primary care (REEACT trial): large scale pragmatic randomised controlled trial. BMJ. 2015;351:h5627.

10. Littlewood E, Duarte A, Hewitt C, Knowles S, Palmer S, Walker S, et al. A randomised controlled trial of computerised cognitive behaviour therapy for the treatment of depression in primary care: the Randomised Evaluation of the Effectiveness and Acceptability of Computerised Therapy (REEACT) trial. Health Technology Assessment 2015;19(101).

11. Hill AM, McPhail SM, Waldron N, Etherton-Beer C, Ingram K, Flicker L, et al. Fall rates in hospital rehabilitation units after individualised patient and staff education programmes: a pragmatic, stepped-wedge, cluster-randomised controlled trial. Lancet. 2015;385(9987):2592-9.

12. Holcomb JB, Tilley BC, Baraniuk S, Fox EE, Wade CE, Podbielski JM, et al. Transfusion of plasma, platelets, and red blood cells in a 1:1:1 vs a 1:1:2 ratio and mortality in patients with severe trauma: the PROPPR randomized clinical trial. JAMA. 2015;313(5):471-82.

13. Baraniuk S, Tilley BC, del Junco DJ, Fox EE, van Belle G, Wade CE, et al. Pragmatic Randomized Optimal Platelet and Plasma Ratios (PROPPR) Trial: design, rationale and implementation. Injury. 2014;45(9):1287-95.

14. Zhu H, Fox EE, Baraniuk S, Holcomb JB, Wade CE. Assessing protocol adherence in a clinical trial with ordered treatment regimens: Quantifying the pragmatic, randomized optimal platelet and plasma ratios (PROPPR) trial experience. Injury. 2016;47(10):2131-7.

15. Honkoop PJ, Loijmans RJ, Termeer EH, Snoeck-Stroband JB, van den Hout WB, Bakker MJ, et al. Symptom- and fraction of exhaled nitric oxide-driven strategies for asthma control: A cluster-randomized trial in primary care. Journal of Allergy & Clinical Immunology. 2015;135(3):682-8.e11.

16. Hui AJ, Lau JY, Lam PP, Chui AO, Fan AS, Lam TY, et al. Comparison of colonoscopic performance between medical and nurse endoscopists: a non-inferiority randomised controlled study in Asia. Gut. 2015;64(7):1058-62.

17. Kempe A, Saville AW, Dickinson LM, Beaty B, Eisert S, Gurfinkel D, et al. Collaborative centralized reminder/recall notification to increase immunization rates among young children: a comparative effectiveness trial. JAMA Pediatrics. 2015;169(4):365-73.

18. Knowles CH, Horrocks EJ, Bremner SA, Stevens N, Norton C, O'Connell PR, et al. Percutaneous tibial nerve stimulation versus sham electrical stimulation for the treatment of faecal incontinence in adults (CONFIDeNT): a double-blind, multicentre, pragmatic, parallel-group, randomised controlled trial. Lancet. 2015;386(10004):1640-8.

19. Horrocks EJ, Bremner SA, Stevens N, Norton C, Gilbert D, O’Connell PR, et al. Double-blind randomised controlled trial of percutaneous tibial nerve stimulation versus sham electrical stimulation in the treatment of faecal incontinence: CONtrol of Faecal Incontinence using Distal NeuromodulaTion (the CONFIDeNT trial). Health Technology Assessment. 2015;19(77).

20. Kutner JS, Blatchford PJ, Taylor DH, Jr., Ritchie CS, Bull JH, Fairclough DL, et al. Safety and benefit of discontinuing statin therapy in the setting of advanced, life-limiting illness: a randomized clinical trial.[Erratum appears in JAMA Intern Med. 2015 May;175(5):869; PMID: 25938325]. JAMA Internal Medicine. 2015;175(5):691-700.

21. Lamb SE, Williamson EM, Heine PJ, Adams J, Dosanjh S, Dritsaki M, et al. Exercises to improve function of the rheumatoid hand (SARAH): a randomised controlled trial. Lancet. 2015;385(9966):421-9.

22. Williams MA, Williamson EM, Heine PJ, Nichols V, Glover MJ, Dritsaki M, et al. Strengthening And stretching for Rheumatoid Arthritis of the Hand (SARAH). A randomised controlled trial and economic evaluation. Health Technology Assessment. 2015;19(19).

23. Moreira RC, Mantovani Mde F, Soriano JV. Nursing Case Management and Glycemic Control Among Brazilians With Type 2 Diabetes: Pragmatic Clinical Trial. Nursing Research. 2015;64(4):272-81.

24. Moseley AM, Beckenkamp PR, Haas M, Herbert RD, Lin CW, Team E. Rehabilitation After Immobilization for Ankle Fracture: The EXACT Randomized Clinical Trial. JAMA. 2015;314(13):1376-85.

25. Mouncey PR, Osborn TM, Power GS, Harrison DA, Sadique MZ, Grieve RD, et al. Trial of early, goal-directed resuscitation for septic shock. New England Journal of Medicine. 2015;372(14):1301-11.

26. Mouncey PR, Osborn TM, Power GS, Harrison DA, Sadique MZ, Grieve RD, et al. Protocolised Management In Sepsis (ProMISe): a multicentre randomised controlled trial of the clinical effectiveness and cost-effectiveness of early, goal-directed, protocolised resuscitation for emerging septic shock. Health Technology Assessment. 2015;19(97).

27. Noto MJ, Domenico HJ, Byrne DW, Talbot T, Rice TW, Bernard GR, et al. Chlorhexidine bathing and health care-associated infections: a randomized clinical trial. JAMA. 2015;313(4):369-78.

28. Perkins GD, Lall R, Quinn T, Deakin CD, Cooke MW, Horton J, et al. Mechanical versus manual chest compression for out-of-hospital cardiac arrest (PARAMEDIC): a pragmatic, cluster randomised controlled trial. Lancet. 2015;385(9972):947-55.

29. Gates S, Lall RS, Quinn T, Deakin CD, Cooke M, Horton J, et al. Prehospital randomised assessment of a mechanical compression device in out-of-hospital cardiac arrest (PARAMEDIC): a pragmatic, cluster randomised trial and economic evaluation. Health Technology Assessment. 2017;21(11):1-176.

30. Rangan A, Handoll H, Brealey S, Jefferson L, Keding A, Martin BC, et al. Surgical vs nonsurgical treatment of adults with displaced fractures of the proximal humerus: the PROFHER randomized clinical trial. JAMA. 2015;313(10):1037-47.

31. Handoll H, Brealey S, Rangan A, Keding A, Corbacho B, Jefferson L, et al. The ProFHER (PROximal Fracture of the Humerus: Evaluation by Randomisation) trial - a pragmatic multicentre randomised controlled trial evaluating the clinical effectiveness and cost-effectiveness of surgical compared with non-surgical treatment for proximal fracture of the humerus in adults. Health Technology Assessment. 2015;19(24).

32. Sackley CM, Walker MF, Burton CR, Watkins CL, Mant J, Roalfe AK, et al. An occupational therapy intervention for residents with stroke related disabilities in UK care homes (OTCH): cluster randomised controlled trial. BMJ. 2015;350:h468.

33. Sackley CM, Walker MF, Burton CR, Watkins CL, Mant J, Roalfe AK, et al. An Occupational Therapy intervention for residents with stroke-related disabilities in UK Care Homes (OTCH): cluster randomised controlled trial with economic evaluation. Health Technology Assessment. 2016;20(15).

34. Scott DL, Ibrahim F, Farewell V, O'Keeffe AG, Walker D, Kelly C, et al. Tumour necrosis factor inhibitors versus combination intensive therapy with conventional disease modifying anti-rheumatic drugs in established rheumatoid arthritis: TACIT non-inferiority randomised controlled trial. BMJ. 2015;350:h1046.

35. Scott DL, Ibrahim F, Farewell V, O'Keeffe AG, Ma M, Walker D, et al. Randomised controlled trial of tumour necrosis factor inhibitors against combination intensive therapy with conventional disease-modifying antirheumatic drugs in established rheumatoid arthritis: the TACIT trial and associated systematic reviews. Health Technology Assessment. 2014;18(66).

36. Semler MW, Weavind L, Hooper MH, Rice TW, Gowda SS, Nadas A, et al. An Electronic Tool for the Evaluation and Treatment of Sepsis in the ICU: A Randomized Controlled Trial. Critical Care Medicine. 2015;43(8):1595-602.

37. Smith JE, Rockett M, S SC, Squire R, Hayward C, Ewings P, et al. PAin SoluTions In the Emergency Setting (PASTIES)--patient controlled analgesia versus routine care in emergency department patients with pain from traumatic injuries: randomised trial. BMJ. 2015;350:h2988.

38. Smith JE, Rockett M, Creanor S, Squire R, Hayward C, Ewings P, et al. PAin SoluTions In the Emergency Setting (PASTIES)—patient controlled analgesia versus routine care in emergency department patients with non-traumatic abdominal pain: randomised trial. bmj. 2015;350:h3147.

39. Stewart S, Ball J, Horowitz JD, Marwick TH, Mahadevan G, Wong C, et al. Standard versus atrial fibrillation-specific management strategy (SAFETY) to reduce recurrent admission and prolong survival: pragmatic, multicentre, randomised controlled trial. Lancet. 2015;385(9970):775-84.

40. Wechsler ME, Yawn BP, Fuhlbrigge AL, Pace WD, Pencina MJ, Doros G, et al. Anticholinergic vs Long-Acting beta-Agonist in Combination With Inhaled Corticosteroids in Black Adults With Asthma: The BELT Randomized Clinical Trial. JAMA. 2015;314(16):1720-30.

41. Westendorp WF, Vermeij JD, Zock E, Hooijenga IJ, Kruyt ND, Bosboom HJ, et al. The Preventive Antibiotics in Stroke Study (PASS): a pragmatic randomised open-label masked endpoint clinical trial. Lancet. 2015;385(9977):1519-26.

42. Williamson I, Vennik J, Harnden A, Voysey M, Perera R, Kelly S, et al. Effect of nasal balloon autoinflation in children with otitis media with effusion in primary care: an open randomized controlled trial. CMAJ Canadian Medical Association Journal. 2015;187(13):961-9.

43. Williamson I, Vennik J, Harnden A, Voysey M, Perera R, Breen M, et al. An open randomised study of autoinflation in 4- to 11-year-old school children with otitis media with effusion in primary care. Health Technology Assessment. 2015;19(72).
