# Supplementary material for: Process evaluation within pragmatic randomised controlled trials: what is it, why is it done, and can we find it?—a systematic review
Source: Trials. 2020 Nov 9;21:916. doi: 10.1186/s13063-020-04762-9 (PMC7650157; doi:10.1186/s13063-020-04762-9)
Supplement: Supplementary file 5 — Additional file 5. Items mapped to each process evaluation component. [file 13063_2020_4762_MOESM5_ESM.docx]

**Additional file 5**

**Items mapped to each process evaluation component**

| **MRC component** | **Included items** |
| --- | --- |
| **Implementation** | |
| **Adaptations**  *Alterations made to an intervention in order to achieve better contextual fit* | Trial papers   - Time taken to deliver interventions where this was not specified by a protocol - Means through which the intervention was delivered where this was flexible, e.g. qualifications of staff delivering the intervention - Which intervention components were delivered to participants as part of flexible interventions - Description of alternative materials used by sites to trial materials   Process evaluation papers   - Description of how a flexible intervention was delivered in practice |
| **Dose**  *How much intervention is delivered* | Trial papers   - Numbers of intervention sessions delivered to participants - Numbers of ‘occurrences’ of optional intervention components delivered to participants - Numbers of times the intervention electronic tool was opened - Time spent by deliverers on intervention components |
| **Fidelity**  *The consistency of what is implemented with the planned intervention* | Trial papers   - Whether or not intervention components were delivered - The quality or standard of (components of) interventions delivered - Reasons for non-adherence or protocol deviations - Fidelity scores, adherence percentages - Whether or not the correct randomised intervention was delivered - Analyses to examine the effect of non-fidelity on the primary outcome – e.g. per-protocol, complier average causal effect analyses   Process evaluation papers   - Whether and how centres delivered interventions in accordance with intervention protocols |
| **How delivery is achieved**  *The structures, resources and mechanisms through which delivery is achieved* | Process evaluation papers   - Qualitative exploration of perceptions of intervention deliverers - Measures taken to ensure fidelity to intervention and usual care protocols |
| **Reach**  *Extent to which target audience comes into contact with intervention* | Trial papers   - Trial flow diagrams / CONSORT diagrams - Reasons for non-participation, exclusion, drop-out - Participant and site characteristics - Numbers of participants recruited from different sites - Numbers of participants who received the randomised intervention - Comparison of demographics between those who declined participation and trial participants - Characteristics of screened but not randomised patients - Reach of interventions delivered to randomised populations - Comparison of demographics between participants completing and not completing follow-up - Comparison of site characteristics with all departments in the country - Comparison of participant characteristics with national patient population - Length of time sites open to recruitment, length of time between obtaining site NHS permission and opening to recruitment - Independent rating of reasons for patients being judged ineligible by sites - Subgroup analysis comparing outcomes between patients randomised to receive the intervention who answered and did not answer at least one call. - Sample attrition bias - Sensitivity analysis of primary outcome including participants with missing outcomes - Sensitivity analysis excluding participants from 2 poorly recruiting centres - Associations between participant characteristics and the completeness of response to providing follow-up data   Process evaluation papers   - Interviews with healthcare professionals about the degree to which they targeted recruitment to patients deemed most suitable, and perceptions about which patients were most suitable for the intervention. - Patient motivations for agreeing or declining trial participation - Measures taken to ensure inclusion of intended trial population in pragmatic trial |
| **Context** | |
| **Causal mechanisms that act to maintain the status quo, or enhance effects** | Trial papers   - Details of usual care received by participants - Use of similar interventions by usual care group, impact of use on outcomes - Change in medication use by trial participants during the intervention period - Impact of concurrent interventions - Seasonal effects   Process evaluation papers   - Participant reported barriers and facilitators to engaging with or adhering to the intervention |
| **Contextual factors that shape theory of how the intervention works** | Trial papers   - Effect of time on effectiveness of the intervention – e.g. cumulative unit level effect of intervention, learning curve effects - Effect of intervention variables e.g. phone calls by answering machine or in person - Ceiling effect of intervention depending on participant baseline level of disability - Comparison of outcomes between participants who kept taking same regime and those who switched partway through   Process evaluation papers   - Qualitative findings discussing potential factors influencing intervention outcomes e.g. skills, experience, personalities and abilities of intervention deliverers |
| **Contextual moderators**  *Shape, and may be shaped by, implementation, intervention mechanisms, and outcomes* | Trial papers   - Analyses of effect of moderators on outcomes, e.g. participant age, gender, smoking status, cognition, treatment preferences, site characteristics   Process evaluation papers   - In qualitative studies – findings about factors which could potentially modify intervention effect |
| **Mechanisms of impact** | |
| **Mediators**  *Intermediate processes which explain subsequent changes in outcome* | Trial papers   - Effect of participant usage of different intervention components on primary outcome   Process evaluation papers   - Mediation analysis of proximal intervention effects |
| **Participant responses**  *How participants interact with a complex intervention* | Trial papers   - Uptake and use of the intervention, or components of the intervention, by trial participants, e.g. number of sessions attended - Analyses to examine the effect of adherence to or completion of an intervention or its components on the primary outcome - Subgroup analyses to investigate the effect of certain participant characteristics on level compliance with intervention - Participant satisfaction with treatment - Participant perceptions of which treatment they had received, treatment preferences at end of trial - Procedure acceptability to participants - Process-of-care outcome e.g. medication adherence, accessing therapies   Process evaluation papers   - Qualitative research exploring patient adherence, perceptions, experiences of interventions - Quantitative questionnaire about participant perceptions of the benefits and harms of the intervention |
| **Unintended pathways and consequences** | Trial papers   - Participant adverse events   Process evaluation papers   - Qualitative findings included reports of unanticipated consequences |
