# Supplementary material for: Process evaluation within pragmatic randomised controlled trials: what is it, why is it done, and can we find it?—a systematic review
Source: Trials. 2020 Nov 9;21:916. doi: 10.1186/s13063-020-04762-9 (PMC7650157; doi:10.1186/s13063-020-04762-9)
Supplement: Supplementary file 6 — Additional file 6. All extracted values of process evaluation. [file 13063_2020_4762_MOESM6_ESM.docx]

**Additional file 6**

**All extracted values of process evaluation**

| **Value category with details** | **Process evaluations reporting this value (n=17)** |
| --- | --- |
| **Adding value to the intervention** | |
| **Supporting implementation of the intervention into practice**   - Targeting or tailoring the intervention to specific patients - Aiding replication of a complex intervention - Understanding how patients engage with the intervention - Understanding providers’ viewpoints and willingness to collaborate - Developing tools / strategies for implementation - Targeting the intervention to specific groups - Highlighting important components of the intervention to implementers - Highlighting benefits of the intervention to promote uptake - Highlighting effective delivery strategies - Providing evidence of feasibility / acceptability - Tailoring delivery to different groups - Highlighting importance of roles of different people / agencies in ensuring successful delivery - Addressing barriers to implementation or uptake of the intervention - Recommendations for training or support to participants or deliverers - Suggesting how intervention could fit into existing care pathways - Highlighting potential disadvantages of the intervention - Recommendations for information to give to patients considering intervention - Recommendations for clinicians to help decide between interventions - Recommendations for further intervention implementation research - Highlighting lack of equipoise in deliverers | 15 |
| **Improving the intervention**  Recommendations for further development of the intervention based on process evaluation findings:   - Recommendations to keep all components of the intervention - Adding stronger monitoring protocols to promote adherence - Adaptations to design for patients with reduced cognition   Recommendations for further research relating to the intervention:   - Effectiveness over time - Effectiveness in different contexts - Different modes of delivery e.g. group settings - Intervention refinement, e.g. to improve patient experience | 10 |
| **Addressing a concern identified about the intervention**   - Acceptability of the intervention to patients / deliverers - Participant adherence - Complexity of intervention delivery - Influence of participant cognition on intervention effectiveness | 7 |
| **Understanding how the intervention works**   - Intervention mechanisms - Content delivered in a flexible intervention | 4 |
| **Adding value to the RCT** | |
| **Providing reasons for trial results**   - Possible reasons for non-positive trial results - Explanations for positive trial results - Explanations for other trial data | 8 |
| **Adding information not provided by the trial**   - Participant or deliverer concerns - Key components of intervention - Added clarification, nuance, context - Perspectives of participants after time for reflection - Concurrent treatments received by trial participants - Experiences and perceptions – things important to participants, minority views | 6 |
| **Increasing accuracy of trial results**   - Assessing comparability of standard care between both randomised groups - Qualitative findings helping confirm quantitative data on satisfaction - Avoid survivor bias - Accurately define the trial population and facilitate purpose and interpretation of trial - Investigating threats to internal and external validity | 6 |
| **Building on trial data**   - Explore findings from a subgroup analysis conducted in the main trial - Expand on the quantitative questionnaire data collected in the main trial about participant acceptability and satisfaction - Identified adverse events not reported in the main trial data collection | 3 |
| **Understanding the applicability of trial results**   - Evaluating whether the intended pragmatic trial population was achieved in the trial - Investigating threats to external validity from patient or provider treatment preference | 2 |
| **Meeting trial reporting requirements**   - Meeting CONSORT requirements for pragmatic and nonpharmacologic trials | 1 |
| **Meeting recommendation to conduct process evaluation**   - Citing recommendation by MRC process evaluation framework to conduct mediation analysis | 1 |
| **Explaining issues with trial conduct**   - Reasons for requiring recruitment extension | 1 |
| **Adding value external to the intervention or RCT** | |
| **Contributing to wider knowledge**   - Future trial design - Understanding patient populations and patient experiences - Understanding the problem addressed by the intervention - Improving clinical practice in the field - Informing design of similar interventions - Highlighting that findings supported or refuted the existing knowledge base - Methodological recommendations | 16 |
| **Improving usual care at trial sites**   - Highlighting gaps in current care provision | 1 |
